# Synthesis, characterization, molecular docking evaluation, antiplatelet and anticoagulant actions of 1,2,4 triazole hydrazone and sulphonamide novel derivatives

**DOI:** 10.1186/s13065-018-0378-5

**Published:** 2018-02-07

**Authors:** Waseem Khalid, Amir Badshah, Arif-ullah Khan, Humaira Nadeem, Sagheer Ahmed

**Affiliations:** 1 Riphah Institute of Pharmaceutical Sciences, Riphah International University, Islamabad, Pakistan; 2 Shifa College of Pharmaceutical Sciences, Shifa Tameer-e-Millat University, Islamabad, Pakistan

**Keywords:** 1,2,4-Triazole derivatives, Hydrazone and sulphonamide derivatives, Antiplatelet, Anticoagulant

## Abstract

In the present study, a series of new hydrazone and sulfonamide derivatives of 1,2,4-triazole were synthesized. Initially three 4-substituted-5-(2-pyridyl)-1,2,4-triazole-3-thiones ZE-1(a–c) were treated with ethyl chloroacetate to get the corresponding thioesters ZE-2(a–c), which were reacted with hydrazine hydrate to the respective hydrazides ZE-3(a–c). The synthesized hydrazides were condensed with different aldehydes and p-toluene sulfonylchloride to furnish the target hydrazone derivatives ZE-4(a–c) and sulfonamide derivatives ZE-5(a–c) respectively. All the synthesized compounds were characterized by FTIR, ^1^HNMR, ^13^CNMR and elemental analysis data. Furthermore, the new hydrazone and sulfonamide derivatives ZE-4(b–c) and ZE-5(a–b) were evaluated for their antiplatelet and anticoagulant activities. ZE-4b, ZE-4c, ZE-5a and ZE-5b inhibited arachidonic acid, adenosine diphosphate and collagen-induced platelets aggregation with IC_50_ values of 40.1, 785 and 10.01 (ZE-4b), 55.3, 850.4 and 10 (ZE-4c), 121.6, 956.8 and 30.1 (ZE-5a), 99.9, 519 and 29.97 (ZE-5b) respectively. Test compounds increased plasma recalcification time (PRT) and bleeding time (BT) with ZE-4c being found most effective, which at 30, 100, 300 and 1000 µM increased PRT to 84.2 ± 1.88, 142 ± 3.51, 205.6 ± 5.37 and 300.2 ± 3.48 s and prolonged BT to 90.5 ± 3.12, 112.25 ± 2.66, 145.75 ± 1.60 s (P < 0.001 vs. saline group) respectively. In silico docking approach was also applied to screen these compounds for their efficacy against selected drug targets of platelet aggregation and blood coagulation. Thus in silico, in vitro and in vivo investigations of ZE-4b, ZE-4c, ZE-5a and ZE-5b prove their antiplatelet and anticoagulant potential and can be used as lead molecules for further development. 
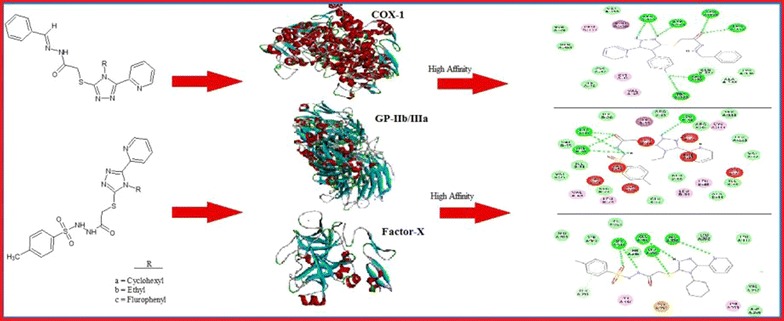

## Introduction

Thrombotic disorders are responsible for major health problems worldwide [1]. According to global burden of diseases, injuries and risk factors study, ischemic heart diseases caused 7.0 million deaths and stroke up to 5.9 million deaths in 2010 only. About 50% of these deaths were caused by thrombosis [2]. Hemostasis maintains normal blood flow in our body and prevents blood loss after vascular injury. Platelet and coagulation factors are essential elements of hemostasis, which are involved in activation and stabilization of thrombin resulting in the formation of thrombus and thus prevention of hemorrhage [3, 4]. Disturbance in normal hemostatic balance or platelet function contributes to development and progression of many thrombotic disorders [5]. There are many antiplatelet and anticoagulant drugs, available commercially, which are being used for the treatment of thrombotic disorders. But these agents are associated with numerous limitations and side effects, including lack of reversibility, a sheer dose response, interactions, narrow therapeutic index, congenital disabilities, miscarriage and most commonly bleeding complications [6, 7]. Therefore, identifying target specific novel antiplatelet and anticoagulant agents with a better efficacy and least side effects is a challenging task for researchers.

**Fig. 1:**
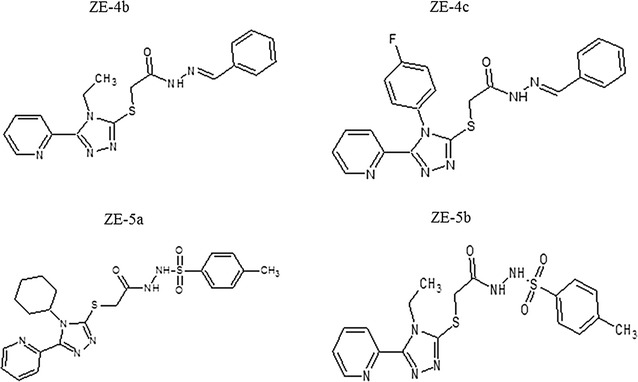
Structures of compounds: *N*-[{(2-phenyl)methylidene]-2-(4-ethyl-5-(pyridine-2-yl)-4*H*-1,2,4-triazole-3-yl)sulfanyl}acetohydrazide (ZE-4b), *N*-[{(2-phenyl)methylidene]-2-(4-(fluorophenyl-5-(pyridine-2-yl)-4*H*-1,2,4-triazole-3yl)sulfanyl}acetohydrazide (ZE-4c), *N*-[{(4-methylphenyl)sulfonyl}]-2-(4-cyclohexyl-5-(pyridine-2-yl)-4*H*-1,2,4-triazole-3-yl)sulfanyl}acetohydrazide (ZE-5a) and *N*-[{(4-methylphenyl)sulfonyl}-2-(4-ethyl-5-(pyridine-2-yl)-4*H*-1,2,4-triazole-3yl)sulfanyl}acetohydrazide (ZE-5b)

Triazole is a five-membered heterocyclic compound with two isomeric forms, i.e. 1,2,3-triazole and 1,2,4-triazole. 1,2,4-Triazoles especially have received much attention as their intriguing physical and biological properties, as well as their excellent stability, rendering them potential drug core structures. Triazole derivatives have wide pharmacological spectrum such as antimicrobial, anti-inflammatory, analgesic, antimalarial, antiviral, antiproliferative, anticancer and various other activities [8]. In a recent study, 1,2,3-triazole derivatives have also shown significant inhibitory activity against blood platelet aggregation and coagulation [9]. Hydrazone is a class of organic compounds having azomethine group R_1_R_2_C=NNH_2_, which are known to possess different pharmacological activities like antimicrobial, analgesic, anti-inflammatory, anticonvulsant, antidiabetic, antitumor and antiplatelet activities [10]. Similarly, sulfonamides are well known class of compounds associated with broad range of activities including antibacterial, anti-inflammatory, carbonic anhydrase inhibitor, hypoglycemic activity, anti-HIV, anticancer and antiplatelet activities [11]. In view of the great importance of triazole, hydrazone and sulfonamide moieties in medicinal chemistry, we would like to report the synthesis of some new hydrazone and sulfonamide derivatives of 4,5-disubstituted-1,2,4-triazole-3-thiones ZE-4(a–c) and ZE-5(a–c). ZE is the structural code given to the synthesized compounds. The synthesized derivatives ZE-4(b–c) and ZE-5(a–b), as shown in Fig. 1, were investigated for their antiplatelet and anticoagulant effects using in vitro and in vivo assays. In addition to this, molecular docking study of synthesized compounds was also performed against selected targets of platelet aggregation and blood coagulation pathways to study the binding interactions which can provide an insight into the possible mechanism of action of these new molecules.

## Materials and methods

### Chemicals

Benzaldehyde, dimethyl sulfoxide, ethanol, ethyl chloroacetate, potassium hydroxide (KOH), *p*-toluene-sulphonyl-chloride were obtained from Merck Millipore., Billerica, MA, USA. Aspirin, calcium chloride (CaCl_2_), diethyl ether, heparin, phosphate buffers solution (PBS), sodium citrate from Sigma chemicals., Dt. Louis, MO, USA. Adenosine diphosphate (ADP), arachidonic acid (AA) and collagen were purchased from Chrono-log association, Havertown, PA, USA.

### Animals

Balb-C mice (25–30 g) of either sex were used, housed at animal house of Riphah Institute of Pharmaceutical Sciences (RIPS) under standard laboratory protocols; at 25 ± 2 °C, duration of light and darkness was set for 12 h each. Mice were given free access to standard diet and water ad libitum. The study performed complied with rules of Institute of Laboratory Animal Resources, Commission on Life Sciences University, National Research Council (1996), approved by RIPS Ethical Committee (Reference No: REC/RIPS/2016/008).

### Chemistry

**Scheme 1:**
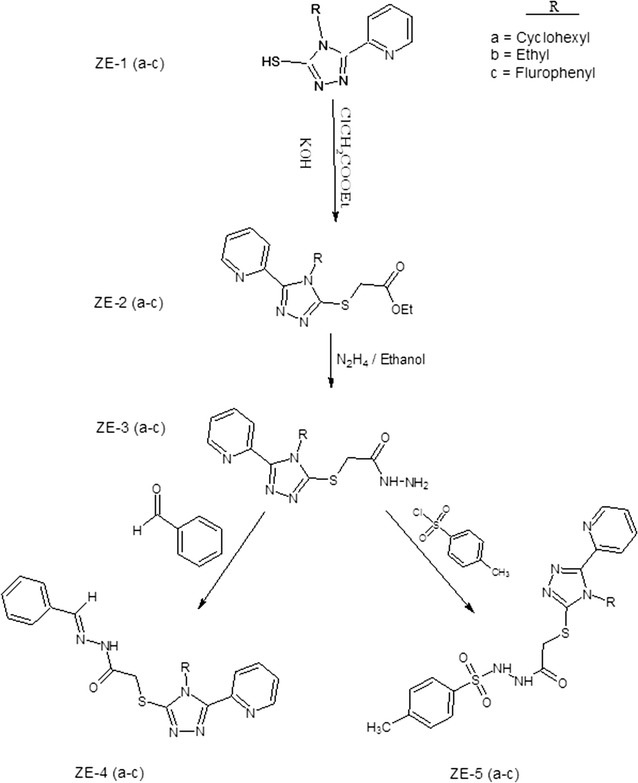
Synthesis of 1,2,4-triazole hydrazone and 1,2,4-triazole sulphonamide derivatives: *N*-[{(2-phenyl)methylidene]-2-(4-cyclohexyl-5-(pyridine-3-yl)-4*H*-1,2,4-triazol-3-yl)sulfanyl}acetohydrazide (ZE-4a), *N*-[{(2-phenyl)methylidene]-2-(4-ethyl-5-(pyridine-2-yl)-4*H*-1,2,4-triazole-3-yl)sulfanyl}acetohydrazide (ZE-4b), *N*-[{(2-phenyl)methylidene]-2-(4-(fluorophenyl-5-(pyridine-2-yl)-4*H*-1,2,4-triazole-3yl)sulfanyl}acetohydrazide (ZE-4c), *N*-[{(4-methylphenyl) sulfonyl}]-2-(4-cyclohexyl-5-(pyridine-2-yl)-4*H*-1,2,4-triazole-3-yl)sulfanyl}acetohydrazide (ZE-5a), *N*-[{(4-methylphenyl) sulfonyl}-2-(4-ethyl-5-(pyridine-2-yl)-4*H*-1,2,4-triazole-3yl)sulfanyl}acetohydrazide (ZE-5b) and *N*-{(4-methylphenyl)sulfonyl]-2-(4-(4-flurophenyl-5-(pyridine-2-yl)-4*H*-1,2,4-triazol-3yl)sulfanyl}acetohydrazide (ZE-5c)

All chemicals were purchased from commercial suppliers and used without further purification. Melting points were determined on a Gallenkamp melting point apparatus and were uncorrected. The IR spectra were recorded on Thermo scientific NICOLET IS10 spectrophotometer. All ^1^HNMR and ^13^CNMR spectra were recorded on Bruker AM-400 spectrophotometer at 400 and 100 MHz respectively, in DMSO as a solvent and TMS as an internal standard. Elemental analyses were performed with a LECO-183 CHN analyzer. 1,2,4-Triazole hydrazone and sulphonamide derivatives were synthesized in three steps, following Scheme 1.

#### Synthesis of 5-(substituted)-1,2,4-triazole-2-thiones ZE-1(a–c)

All the substituted mercapto triazoles ZE-1(a–c) were synthesized previously by the reported procedure. The triazoles were characterized by comparing their melting points with the reported literature [12].

#### Synthesis of 1,2,4-triazole esters ZE-2(a–c)

0.003 mol of respective triazoles ZE-1(a–c) were dissolved in 50 mL of absolute ethanol and a solution of 0.003 mol (0.168 g) of KOH in 20 mL of water was added dropwise to the mixture with continuous stirring. After 30-min, ethyl chloroacetate was slowly added to the reaction mixture and refluxed for 2–3 h. The progress of the reaction was monitored by thin layer chromatography (TLC) (ethyl acetate: petroleum ether 2:1). After completion of the reaction, the solvent was evaporated in vacuo and the crude product thus obtained was recrystallized from ethanol to get the corresponding triazole thioesters ZE-2(a–c) [12, 13].

##### *Ethyl [{4*-*cyclohexyl*-*5*-*(pyridine*-*2*-*yl)*-*4H*-*1,2,4*-*triazol*-*3*-*yl]sulfanyl}acetate* (ZE-2a)

Yield 78%, M.P. 147–149 °C, R_f_ 0.77 (ethyl acetate: pet. ether 2:1); IR (KBr) cm^−1^: 2972 (C–H), 1726 (C=O, ester), 1665 (C=N), 1505 (C=C); ^1^H-NMR (DMSO-d_6_, 400 MHz): δ 8.60 (d, 1H, J = 7.6 Hz, Py H-3), 8.01 (d, 1H, J = 7.9, Py H-6), 7.80 (t, 1H, J = 7.8 Hz, Py H-4), 7.36 (dd, 1H, J = 7.6 Hz, J = 7.8 Hz, Py H-5), 4.45 (m, 1H, cyclohexyl H-1), 4.12 (s, 2H, CH_2_–S), 3.16 (q, 2H, J = 7.0 Hz, OCH_2_), 1.31 (t, 3H, J = 6.9 Hz, CH_3_), 1.25–1.81 (m, 10H, cyclohexyl H). ^13^CNMR (DMSO-d_6_, 100 MHz): δ 167.8 (C=O), 152.5, 146.3, 145.6, 143.2, 135.4, 123.3, 120.4, 62.1, 58.3, 57.2, 30.6, 29.8 (2C), 25.4 (2C), 24.9, 13.8. Anal. Calcd. For C_17_H_22_N_4_O_2_S: C, 58.95; H, 6.35; N, 16.18.

Found: C, 58.56; H, 6.40; N, 16.27.

##### *Ethyl [{4*-*ethyl*-*5*-*(pyridine*-*2*-*yl)*-*4H*-*1,2,4*-*triazol*-*3*-*yl]sulfanyl}acetate* (ZE-2b)

Yield 81%, M.P. 155–157 °C, R_f_ 0.81 (ethyl acetate: petroleum ether 2:1); IR (KBr) cm^−1^: 2985 (C–H), 1730 (C=O, ester), 1625 (C=N) 1446 (C=C); ^1^HNMR (DMSO-d_6_, 400 MHz): δ 8.71 (d, 1H, J = 7.6 Hz, Py H-3), 8.05 (d, 1H, J = 7.9 Hz, Py H-6), 8.01 (t, 1H, J = 7.6 Hz, Py H-4), 7.41 (dd, 1H, J_4,5_ = 7.5 Hz, J_5,6_ = 7.9 Hz, Py H-5), 4.50 (q, 2H, J = 6.9 Hz, CH_2_), 4.29 (s, 2H, CH_2_–S), 3.67 (q, 2H, J = 6.8 Hz, OCH_2_), 1.33 (t, 3H, J = 7.0 Hz, CH_3_), 1.30 (t, 3H, J = 6.7 Hz, CH_3_). ^13^CNMR (DMSO-d_6_, 100 MHz): δ 166.7 (C=O), 153.1, 147.2, 146.6, 145.4, 134.8, 122.7, 121.3, 61.8, 42.5, 32.5, 13.2, 12.1. Anal. Calcd. For C_13_H_16_N_4_O_2_S: C, 53.42; H, 5.47; N, 19.17.

Found: C, 53.40; H, 5.39; N, 19.10.

##### *Ethyl [{4*-*(4*-*flurophenyl)*-*5*-*(pyridine*-*2*-*yl)*-*4H*-*1,2,4*-*triazol*-*3*-*yl]sulfanyl}acetate* (ZE-2c)

Yield 78%, M.P. 252–260 °C, R_f_ 0.79 (ethyl acetate: petroleum ether 2:1);IR (KBr) cm^−1^: 2985 (C–H), 1735 (C=O, ester), 1607 (C=N),1510 (C=C); ^1^H-NMR (DMSO-d_6_, 400 MHz): δ 8.39 (d, 1H, J = 7.7 Hz, Py H-3), 8.00 (d, 1H, J = 7.8 Hz, Py H-6), 7.60 (t, 1H, J = 7.6 Hz, Py H-4), 7.36 (dd, 1H, J_4,5_ = 7.5, J_5,6_ = 7.6 Hz, Py H-5), 7.26–7.31 (m, 4H, Ar–H), 4.33 (s, 2H, CH_2_–S), 3.41 (q, 2H, J = 6.9 Hz, OCH_2_), 1.27 (t, 3H, J = 6.7 Hz, CH_3_). ^13^CNMR (DMSO-d_6_, 100 MHz): δ 166.7 (C=O), 160.1 (C–F), 152.6, 147.3, 146.2, 145.0, 143.7, 136.3, 124.8 (2C), 123.6, 122.7, 115.6 (2C), 60.8, 32.6, 13.8. Anal. Calcd. For C_17_H_15_N_4_O_2_SF: C, 56.98; H, 4.18; N, 15.64.

Found: C, 56.96; H, 4.15; N, 15.39.

#### Synthesis of 1,2,4-triazolehydrazides ZE-3(a–c)

A mixture of 0.002 mol of respective triazole esters ZE-2(a–c) and 0.006 mol of hydrazine hydrate in absolute ethanol was refluxed for 4–5 h with stirring. The progress of the reaction was monitored by TLC (ethyl acetate: petroleum ether 2:1). After completion, the reaction mixture was allowed to cool and excess hydrazine was evaporated. The crude solid was filtered off and recrystallized from ethanol to give the corresponding hydrazides ZE-3(a–c) [14].

##### *2*-*[{4*-*Cyclohexyl*-*5*-*(pyridine*-*2*-*yl)*-*4H*-*1,2,4*-*triazol*-*3*-*yl]sulfanyl}acetohydrazide* (ZE-3a)

Yield 68%, M.P. 143–145 °C, R_f_ 0.78 (ethyl acetate: petroleum ether 2:1); IR (KBr) cm^−1^: 3347 (N–H), 2985 (C–H), 1687 (C=O, amide), 1650 (C=N), 1448 (C=C); ^1^HNMR (DMSO-d_6_, 400 MHz): δ 9.23 (s, 1H, NH), 8.75 (d, 1H, J = 7.4 Hz, Py H-3), 8.01 (d, 1H, J = 7.8 Hz, J = 5.2 Hz, Py H-6), 7.82 (t, 1H, J = 7.6 Hz, Py H-4), 7.26 (dd, 1H, J = 7.5 Hz, J = 5.4 Hz, Py H-5), 4.97 (s, 1H, NH_2_), 4.56 (m, 1H, cyclohexyl H-1), 4.32 (s, 2H, CH_2_–S), 1.26–1.81 (m, 10H, cyclohexyl H). ^13^CNMR (DMSO-d_6_, 100 MHz): δ 164.5 (C=O), 152.6, 146.8, 144.6, 143.2, 138.4, 123.3, 120.4, 56.3, 29.8, 29.2 (2C), 25.4 (2C), 24.9. Anal. Calcd. For C_15_H_20_N_6_OS: C, 54.21; H, 6.02; N, 25.30.

Found: C, 54.06; H, 6.01; N, 25.10.

##### *2*-*[{4*-*Ethyl*-*5*-*(pyridine*-*2*-*yl)*-*4H*-*1,2,4*-*triazol*-*3*-*yl]sulfanyl}acetohydrazide* (ZE-3b)

Yield 76%, M.P. 147–148 °C, R_f_ 0.80 (ethyl acetate: petroleum ether 2:1); IR (KBr) cm^−1^: 3270 (N–H), 2991 (C–H), 1670 (C=O, amide), 1623 (C=N), 1417 (C=C); ^1^HNMR (DMSO-d_6_, 400 MHz): δ 9.47 (s, 1H, NH), 8.74 (d, 1H, J = 7.7 Hz, Py H-3), 8.03 (d, 1H, J = 7.9 Hz, Py H-6), 7.83 (t, 1H, J = 7.5 Hz, Py H-4), 7.28 (dd, 1H, J = 7.5 Hz, J = 7.8 Hz, Py H-5), 5.25 (s, 2H, NH_2_) 4.38 (s, 2H, CH_2_–S), 4.19 (q, 2H, J = 6.7 Hz, CH_2_), 1.32 (t, 3H, J = 6.9 Hz, CH_3_). ^13^CNMR (DMSO-d_6_, 100 MHz): δ 164.7 (C=O), 153.1, 147.2, 146.6, 145.4, 134.8, 123.7, 121.3, 41.3, 30.5, 12.8. Anal. Calcd. For C_11_H_14_N_6_OS: C, 47.48; H, 5.03; N, 30.21. Found: C, 47.50; H, 5.00; N, 30.13.

##### *2*-*[{4*-*(4*-*Flurophenyl)*-*5*-*(pyridine*-*2*-*yl)*-*4H*-*1,2,4*-*triazol*-*3*-*yl]sulfanyl}acetohydrazide* (ZE-3c)

Yield 71%, M.P. 241–242 °C, R_f_ 0.69 (ethyl acetate: petroleum ether 2:1); IR (KBr) cm^−1^: 3234 N–H), 2965 (C–H), 1665 (C=O, amide), 1627 (C=N), 1423 (C=C); ^1^H NMR (DMSO-d_6_, 400 MHz) δ 9.91 (s, 1H, N–H), 8.65 (d, 1H, J = 7.3 Hz Py H-3), 8.04 (d, 1H, J = 6.7 Hz, Py H-6), 7.81 (t, 1H, J = 7.3 Hz, Py H-4), 7.38 (dd, 1H, J = 7.2 Hz, J = 6.6 Hz, Py H-5), 7.22–7.28 (m, 4H, Ar–H), 5.10 (s, 2H, NH_2_), 4.33 (s, 2H, CH_2_–S). ^13^CNMR (DMSO-d_6_, 100 MHz): δ 165.1 (C=O), 160.4 (C–F), 152.8, 148.6, 147.9, 144.0, 143.7, 136.3, 125.5 (2C), 123.6, 121.7, 115.6 (2C), 30.6. Anal. Calcd. For C_15_H_13_N_6_OSF: C, 58.95; H, 6.35; N, 16.18. Found: C, 52.32; H, 3.77; N, 24.41.

#### Synthesis of 1,2,4-triazolehydrazones ZE-4(a–c)

Equimolar quantities of respective hydrazide and aromatic aldehydes (6 mmol) were dissolved in ethanol (50 mL) containing 2–3 mL of glacial acetic acid. The reaction mixture was refluxed for 2–3 h until the completion of reaction as monitored by TLC (ethyl acetate: petroleum ether 2:1). After cooling, the reaction mixture was concentrated in vacuo and the solid obtained was recrystallized from ethanol [15].

##### *N*-*[{(2*-*Phenyl)methylidene]*-*2*-*(4*-*cyclohexyl*-*5*-*(pyridine*-*3*-*yl)*-*4H*-*1,2,4*-*triazol*-*3*-*yl)sulfanyl}acetohydrazide* (ZE-4a)

Yield 66%, M.P. 148–150 °C, R_f_ 0.76 (ethyl acetate: petroleum ether 2:1); IR (KBr) cm^−1^: 3390–3215 (NH), 2990 (C–H), 1624 (C=O, amide), 1556 (C=N), 1465 (C=C); ^1^H NMR (DMSO-d_6_, 400 MHz): δ 9.19 (s, 1H, N–H), 8.74 (bs, 1H, N=CH), 8.72 (d, 1H, J = 7.2 Hz, Py H-3), 8.02 (d, 1H, J = 6.7 Hz, Py H-6), 7.99 (t, 1H, J = 7.3 Hz, Py H-4), 7.94 (dd, 1H, J = 7.1 Hz, J = 6.7 Hz, Py H-5), 7.50–756 (m, 4H, Ar–H), 4.22 (m, 1H, cyclohexyl H-1), 4.13 (s, 2H, CH_2_–S), 1.27–1.81 (m, 10H, cyclohexyl H). ^13^CNMR (DMSO-d_6_, 100 MHz): δ 166.4 (C=O), 152.3, 148.6, 147.5, 143.7, 141.8, 136.8, 135.6, 129.0, 128.5 (2C), 127.3 (2C), 123.3, 120.5, 56.8, 32.0, 31.1 (2C), 26.0, 25.2 (2C). Anal. Calcd. For C_22_H_24_N_6_OS: C, 62.85; H, 5.71; N, 20.00. Found: C, 62.54; H, 5.65; N, 19.96.

##### *N*-*[{(2*-*Phenyl)methylidene]*-*2*-*(4*-*ethyl*-*5*-*(pyridine*-*2*-*yl)*-*4H*-*1,2,4*-*triazol*-*3*-*yl)sulfanyl}acetohydrazide* (ZE-4b)

Yield 81%, M.P. 160–162 °C, R_f_ 0.67 (ethyl acetate: petroleum ether 2:1); IR (KBr) cm^−1^: 3375–3237 (N–H), 2989 (C–H), 1637 (C=O, amide), 1575 (C=N), 1498 (C=C); ^1^H NMR (DMSO-d_6_, 400 MHz); δ 9.31 (bs, 1H, NH), 9.10 (s, 1H, N=CH), 8.37 (d, 1H, J = 6.8 Hz, Py H-3), 8.01 (d, 1H, J = 7.5 Hz, Py H-6), 7.72 (t, 1H, J = 6.8 Hz, Py H-4), 7.58 (dd, 1H, J = 6.7 Hz, J = 7.6 Hz, Py H-5), 7.33–7.41 (m, 4H, Ar–H), 4.50 (q, 2H, J = 6.9 Hz, CH_2_), 4.12 (s, 2H, CH_2_–S), 1.29 (t, 3H, J = 6.9 Hz, CH_3_). ^13^CNMR (DMSO-d_6_, 100 MHz): δ 165.8, 150.7, 148.5, 148.3, 143.9, 141.7, 137.3, 135.6, 128.5, 127.6 (2C), 126.9, 122.3, 120.5, 43.8, 32.1, 12.2. Anal. Calcd. For C_18_H_18_N_6_OS: C, 59.01; H, 4.91; N, 22.95. Found: C, 58.96; H, 4.82; N, 22.63.

##### *N*-*[{(2*-*Phenyl)methylidene]*-*2*-*(4*-*(*-*flurophenyl*-*5*-*(pyridine*-*2*-*yl)*-*4H*-*1,2,4*-*triazole*-*3*-*yl)sulfanyl}acetohydrazide* (ZE-4c)

Yield 80%, M.P. 195–198 °C, R_f_ 0.66 (ethyl acetate: petroleum ether 2:1); IR (KBr) cm^−1^: 3385–3225 (N–H), 2985 (C–H), 1617 (C=O, amide), 1590 (C=N), 1469 (C=C); ^1^H-NMR (DMSO-d_6_, 400 MHz): δ 9.35 (bs, 1H, N–H), 9.05 (s, 1H, N=CH), 8.56 (d, 1H, J = 6.8 Hz, Py H-3), 7.91 (t, 4H, J = 7.6 Hz, Py H-6), 7.70 (t, 1H, J = 6.9 Hz, Py H-4), 7.48 (dd, 1H, J = 7.5 Hz, J = 6.8 Hz, Py H-5), 7.35–7.41 (m, 4H, Ar–H), 7.02–7.10 (m, 4H, Ar–H), 4.29 (s, 2H, CH_2_–S). ^13^CNMR (DMSO-d_6_, 100 MHz): δ 165.4 (C=O), 160.2 (C–F), 151.3, 148.4, 148.0, 144.7, 143.7, 142.4, 137.4, 135.6, 128.7, 128.2 (2C), 127.8 (2C), 127.0 (2C), 123.3, 120.6, 115.8 (2C), 32.1. Anal. Calcd. For C_22_H_17_N_6_OSF: C, 61.11; H, 3.93; N, 19.44. Found: C, 61.01; H, 3.95; N, 19.45.

#### Synthesis of 1,2,4-triazole sulphonamides ZE-5(a–c)

To a solution of 0.01 mol of corresponding hydrazides ZE-3(a–e) in ethanol, 0.01 mol of potassium carbonate and 0.01 mol of *p*-toluene sulfonyl chloride were added. The mixture was refluxed with stirring for 2–3 h. The progress of the reaction was checked by TLC (Ethyl acetate: Petroleum ether 2:1). After completion of the reaction, the reaction mixture was cooled and filtered. The filtrate was then acidified to pH of 1–2 with 2 N hydrochloric acid. The solid product separated was filtered and recrystallized from ethanol [16].

##### *N*-*{(4*-*Methylphenyl)sulfonyl]*-*2*-*(4*-*cyclohexyl*-*5*-*(pyridine*-*2*-*yl)*-*4H*-*1,2,4*-*triazol*-*3yl)sulfanyl}acetohydrazide* (ZE-5a)

Yield 83%, M.P. 250–251 °C, R_f_ 0.58 (ethyl acetate: petroleum ether 2:1); IR (KBr) cm^−1^:3337 (N–H), 2985 (C–H), 1660 (C=O, amide), 1568 (C=N), 1404 (C=C), 1384 (O=S=O); ^1^H NMR (DMSO-d_6_, 400 MHz): δ 9.51 (s, 1H, NH), 8.67 (d, 1H, J = 5.9 Hz, Py H-3), 8.01 (d, 1H, J = 7.9 Hz, Py H-6), 7.57 (t, 1H, J = 6.0 Hz, Py H-4), 7.48 (dd, 1H, J = 7.8 Hz, J = 6.2 Hz, Py H-5), 7.11–7.13 (m, 4H, Ar–H), 4.40 (m, 1H, cyclohexyl H-1), 4.16 (s, 2H, CH_2_–S), 2.27 (s, 3H, ArCH_3_), 1.21–1.81 (m, 10H, cyclohexyl H). ^13^CNMR (DMSO-d_6_, 100 MHz): δ 167.3 (C=O), 151.5, 148.2, 147.7, 143.9, 1143.2, 137.9, 137.2, 129.2 (2C), 128.4 (2C), 123.3, 121.1, 56.8, 32.0, 31.1 (2C), 25.8, 25.1 (2C), 20.9. Anal. Calcd. For C_22_H_26_N_6_O_3_S_2_: C, 54.32; H, 5.34; N, 17.28. Found: C, 54.16; H, 5.36; N, 17.15.

##### *N*-*{(4*-*Methylphenyl)sulfonyl]*-*2*-*(4*-*ethyl*-*5*-*(pyridine*-*2*-*yl)*-*4H*-*1,2,4*-*triazol*-*3yl)sulfanyl}acetohydrazide* (ZE-5b)

Yield 85%, M.P. 265–266 °C, R_f_ 0.72 (ethyl acetate: petroleum ether 2:1); IR (KBr) cm^−1^: 3375 (N–H), 2990 (C–H), 1670 (C=O, amide), 1456 (C=C), 1500 (C=N), 1413 (O=S=O); ^1^H NMR (DMSO-d_6_, 400 MHz): δ 9.21 (s, 1H, NH), 8.73 (d, 1H, J = 5.7 Hz, Py H-3), 8.14 (d, 1H, J = 7.6 Hz, Py H-6), 7.97 (t, 1H, J = 5.9 Hz, Py H-4), 7.55 (dd, 1H, J = 7.5 Hz, J = 6.0 Hz, Py H-5), 7.10–7.13 (m, 4H, Ar–H), 4.50 (q, 2H, J = 6.6 Hz, CH_2_), 4.13 (s, 2H, CH_2_–S), 2.29 (s, 3H, ArCH_3_), 1.33 (t, 3H, J = 6.8 Hz, CH_3_). ^13^CNMR (DMSO-d_6_, 100 MHz): δ 166.8 (C=O), 160.1 (C–F), 151.8, 148.6, 147.9, 144.0, 143.4, 137.8, 137.1, 129.2 (2C), 128.3 (2C), 122.8, 120.3, 43.7, 32.1, 21.0, 12.6. Anal. Calcd. For C_18_H_20_N_6_O_3_S_2_: C, 50.00; H, 4.62; N, 19.44. Found: C, 50.04; H, 4.56; N, 19.41.

##### *N*-*{(4*-*Methylphenyl)sulfonyl]*-*2*-*(4*-*(4*-*flurophenyl*-*5*-*(pyridine*-*2*-*yl)*-*4H*-*1,2,4*-*triazole*-*3*-*yl)sulfanyl}acetohydrazide* (ZE-5c)

Yield 61%, M.P. 240–242 °C, R_f_ 0.69 (ethyl acetate: petroleum ether 2:1); IR (KBr) cm^−1^: 3370 (NH), 2991 (C–H), 1675 (C=O, amide), 1446 (C=C), 1497 (C=N), 1408 (O=S=O); ^1^H NMR (DMSO-d_6_, 400 MHz): δ 9.60 (s, 1H, NH), 8.74 (d, 1H, J = 6.7 Hz, Py H-3), 8.01 (d, 1H, J = 7.6 Hz, Py H-6), 7.95 (t, 1H, J = 6.8 Hz, Py H-4), 7.57 (dd, 1H, J = 7.6 Hz, J = 6.9 Hz, Py H-5), 7.48–7.51 (m, 4H, ArH), 7.11–7.13 (m, 4H, ArH), 4.16 (s, 2H, CH_2_–S), 2.33 (s, 3H, ArCH_3_). ^13^CNMR (DMSO-d_6_, 100 MHz): δ 166.8 (C=O), 160.1 (C–F), 151.8, 148.6, 147.9, 144.0, 143.4, 142.8, 137.8, 137.1, 129.2 (2C), 128.0 (2C), 126.2 (2C), 122.8, 120.3, 115.4 (2C), 32.1. Anal. Calcd. For C_22_H_19_N_6_O_3_S_2_F: C, 54.32; H, 3.81; N, 16.86. Found: C, 54.21; H, 3.80; N, 16.69.

### Antiplatelet assay

Antiplatelet activity was determined by whole blood aggregometry method using three different platelet aggregation inducing agonists namely as, A.A, ADP and collagen [17]. Blood samples from healthy volunteers were obtained in clean plastic tubes containing 3.2% sodium citrate anticoagulant (9:1) and were tested subsequently for 30-min to 5-h. The study was performed at 37 °C at stirring speed of 1200 rpm. As per guidelines of the manufacturer, 500 µL of citrated blood was diluted with same volume of normal saline. 30 µL of different concentrations (1, 3, 10, 30, 100, 300 and 1000 µM) of test compounds were added and then warmed at 37 °C in incubation well of aggregometer for 5-min. After placing electrode, aggregation was induced by various stimulatory agonists, like AA (1.5 mM), ADP (10 µM) and collagen (5 µg/mL). Response (platelet aggregation) was recorded up to 6-min as electrical impedance in ohms. From these platelet aggregation values of 3–4 individual experiments, percent mean platelet inhibition was calculated.

### Anticoagulant activity

#### Plasma recalcification time (PRT)

Anticoagulant activity of test compounds was determined by PRT method [18]. The blood samples were obtained from normal healthy volunteers in containers containing 3.8% sodium citrate (9:1) to prevent the clotting process. Platelet poor plasma was obtained by centrifuging the blood samples at 3000 rpm for 15-min. 200 µL plasma, 100 µL of different concentrations (30, 100, 300 and 1000 μM) of ZE-4b, ZE-4c, ZE-5a and ZE-5b and 300 µL of CaCl_2_ (25 mM) were added together in a clean test tube and incubated in a water bath at 37 °C. The clotting time was recorded using stop watch by tilting test tubes every 5–10 s. Heparin (440 μM) was used as positive control [19].

#### Bleeding time (BT)

Anticoagulant potential of test compounds was also assayed by in vivo tail BT method in mice [20]. Briefly, test compounds ZE-4b, ZE-4c, ZE-5a and ZE-5b in 100, 300 and 1000 μg/kg doses were injected intravenously into the tail vein of mice, fasted overnight. After 10-min, mice were anesthetized using diethyl ether and 2–3 mm deep cut was made at their tails. The tail was then immersed into PBS previously warmed to 37 °C. BT was recorded from time when bleeding started to the time when it completely stopped. The recording was made up to 10 min.

### Docking studies

Protein–ligand docking studies were performed with test derivatives ZE-4(b–c) and ZE-5(a–b) using AutoDock software against selected targets of platelet aggregation and blood coagulation. Affinity was determined by the E-value or binding energy value (kcal/mol) of the best pose of the ligand-receptor complex. 3D structures of test compounds were drawn in protein data bank (PDB) format through Biovia Discovery Studio Visualizer client 2016. Test compounds were docked against eleven selected target receptors. Six of them being involved in regulation of platelet aggregation were cyclooxygenase-1 (COX-1), glycoprotein-IIb/IIIa (GPIIb/IIIa), glycoprotein-VI (GP-VI), purino receptor P_2_Y_12_, prostacyclin (PG-I_2_) receptor and protein activated receptor-1 (PAR-1) with PDB-IDs: 3N8X, 2VDM, 2G17, 4PXZ, 4F8K and 3VW7 respectively. The target proteins mediating blood coagulation process are antithrombin III (AT-III), factor-X (F-X), factor-II (F-II), factor-IX (F-IX) and vitamin-K epoxide reductase (VKOR) having PDB-IDs: 2B4X, 1KSN, 5JZY, 1RFN and 3KP9 respectively. These targets were obtained from http://www.rcsb.org/pdb/home/home.do in PDB format which were then purified through “Discovery Studio Visualizer” software. Standard drugs were obtained from https://pubchem.ncbi.nlm.nih.gov/search/search.cgi, in mol format and converted to PDB format via Open Babel JUI software. Reference drugs used for platelet receptors include aspirin (PubChem CID: 2244), tirofiban (PubChem CID: 60947), hinokitiol (PubChem CID: 3611), the active metabolite of clopidogrel (PubChem CID: 10066813), beraprost (PubChem CID: 6917951) and vorapaxar (PubChem CID: 10077130). For blood coagulation receptors, standard drugs used were heparin sulfate (PubChem CID: 53477714), apixaban (PubChem CID: 10182969), argatroban (PubChem CID: 92722), pegnivacogin (PubChem CID: 86278323) and warfarin (PubChem CID: 54678486). Discovery Studio Visualizer was also utilized for post-docking analysis and schematic representation of hydrogen bonds (classical and non-classical), hydrophobic interactions and amino acid residues involved in hydrogen bonding of the best-docked pose of the ligand–protein complex.

### Statistical analysis

Data expressed as a mean ± standard error of mean (SEM) and analyzed by one-way analysis of variance (ANOVA), with post hoc-Tukey’s test. *P* < 0.05 was considered, as significantly different. The bar graphs were analyzed by Graph Pad Prism (GraphPad, San Diego, CA, USA).

## Results

### Chemistry

The synthesis of all the intermediates and target compounds was accomplished by the reaction sequence shown in Scheme 1. Initially, triazole thioacetate ZE-2(a–c) were synthesized by the reaction of corresponding triazoles ZE-1(a–c) with ethyl chloroacetate in the presence of KOH, which were converted to hydrazides ZE-3(a–c) by reaction with hydrazine hydrate. The treatment of acetohydrazides with benzaldehyde produced the corresponding hydrazone derivatives ZE-4(a–c). Also, the intermediate hydrazides were condensed with *p*-toluene sulfonyl chloride to get the sulfonamide derivatives ZE-5(a–c). The purity of all the synthesized compounds was established by thin layer chromatography and elemental analysis data. All compounds yielded a single spot in different solvent systems showing the purity of the product. Compounds were further characterized by FTIR, ^1^HNMR and ^13^CNMR spectroscopy. The IR spectra of ZE-2(a–c) showed a strong C=O stretch of ester at 1728–1732 cm^−1^. Similarly, ^1^HNMR and ^13^CNMR data also confirmed the formation of an ester. A quartet of CH_2_ at 3.57 ppm and a triplet of CH_3_ at 1.33 ppm was observed due to ethyl moiety of ester. The methylene protons attached to sulfur appeared downfield at 4.47 ppm as singlet due to deshielding effect of two electron withdrawing groups. Characteristic peaks corresponding to pyridyl moiety were observed downfield in the expected region. The IR spectra of hydrazides ZE-3(a–c) showed NH stretchings at 3234–3347 cm^−1^ and amide C=O appeared at 1665–1687 cm^−1^ confirming the formation of hydrazides. The^1^HNMR spectra showed two characteristic absorptions (singlet at 9.25–9.91 ppm and 5.10–5.25 ppm) corresponding to NH and NH_2_ protons of hydrazide group. In the ^1^HNMR spectra of ZE-4(a–c) characteristic singlet at 8.7–9.0 ppm was observed due to N=CH of imine moiety. The NH protons resonated downfield at 8.72–9.57 ppm as a broad singlet. Additional signals due to aromatic protons of phenyl group were observed in the range of 7.23–7.37 ppm as multiplet. The pyridyl protons appeared downfield as expected. The sulfonamide derivatives ZE-5-(a–c) were also characterized by their IR and NMR data. The IR spectra showed characteristic absorptions due to O=S=O at 1340–1413 cm^−1^. In the ^1^HNMR data signals for methyl protons of *p*-toluene sulfonyl moiety were observed as singlet at 2.30 ppm. The NH protons appeared downfield as singlets due to deshielding effect of sulfonyl and carbonyl groups. Aromatic protons resonated in the range of 7.33–7.39 ppm. In the ^13^CNMR spectra of all compounds, carbonyl carbon resonated most downfield at 165–168 ppm and methylene carbon attached to sulfur was observed at 31.2–32.6 ppm. Signals corresponding to carbon atoms of triazole moiety were observed at 151–152 and 147–148 ppm. Methine carbon in ZE-4(a–c) resonated at 143–144 ppm. All the other protons appeared in the expected region.

### Antiplatelet assay

#### Inhibitory effect on AA-induced platelet aggregation

The antiplatelet activity of compounds ZE-4(b–c) and ZE-5(a–b) was determined by whole blood aggregometry method using Chrono-Log impedance aggregometer, model 591. The test compounds were used in 1, 3, 10, 30, 100, 300 and 1000 µM concentrations to observe their inhibitory effect. ZE-4b inhibited platelet aggregation to 4.4 ± 0.09, 8.8 ± 0.09, 30.3 ± 0.06, 41.2 ± 0.23, 63.2 ± 0.06, 78 ± 0.14 and 89.5 ± 0.23% respectively with IC_50_ value of 40.1 µM. ZE-4c inhibited platelet aggregation to 7.9 ± 0.15, 15.4 ± 0.20, 29 ± 0.21, 43 ± 0.18, 59 ± 0.03, 75 ± 0.10 and 86.4 ± 0.44% respectively with IC_50_ value of 55.3 µM. The antiplatelet effect of ZE-5a was 4.0 ± 0.12, 7.9 ± 0.06, 23.7 ± 0.15, 39.5 ± 0.21, 47.4 ± 0.12, 68 ± 0.35 and 72.8 ± 0.59% respectively with IC_50_ value of 121.6 µM. Similarly, ZE-5b inhibited platelet aggregation to 8.8 ± 0.09, 11.4 ± 0.27, 25 ± 0.21, 30.7 ± 0.58, 52.2 ± 0.40, 68.4 ± 0.40 and 79 ± 0.60% respectively with IC_50_ value of 99.9 µM. The standard drug aspirin exhibited inhibition of 27.2 ± 0.18, 36 ± 0.09, 50.1 ± 0.16, 59.7 ± 0.09 and 100% respectively with IC_50_ value of 10.01 µM, as presented in Table 1.

**Table 1:** Inhibitory effect of *N*-[{(2-phenyl)methylidene]-2-(4-ethyl-5-(pyridine-2-yl)-4*H*-1,2,4-triazole-3-yl)sulfanyl}acetohydrazide (ZE-4b), *N*-[{(2-phenyl)methylidene]-2-(4-(fluorophenyl-5-(pyridine-2-yl)-4*H*-1,2,4-triazole-3yl)sulfanyl}acetohydrazide (ZE-4c), *N*-[{(4-methylphenyl)sulfonyl}]-2-(4-cyclohexyl-5-(pyridine-2-yl)-4*H*-1,2,4-triazole-3-yl)sulfanyl}acetohydrazide (ZE-5a) and *N*-[{(4-methylphenyl) sulfonyl}-2-(4-ethyl-5-(pyridine-2-yl)-4*H*-1,2,4-triazole-3yl)sulfanyl}acetohydrazide (ZE-5b) on arachidonic acid (AA), adenosine diphosphate (ADP) and collagen induced platelet aggregation

Test sample	Agonists	% inhibition of platelet aggregation							IC_50_ (µM)
		1 µM	3 µM	10 µM	30 µM	100 µM	300 µM	1000 µM	
ZE-4b	AA	4.4 ± 0.09	8.8 ± 0.09	30.3 ± 0.06	41.2 ± 0.23	63.2 ± 0.06	78 ± 0.14	89.5 ± 0.23	40.1
	ADP	0.1 ± 0.03	1.0 ± 0.03	3.6 ± 0.03	9.6 ± 0.06	18.2 ± 0.12	39.4 ± 0.17	54.7 ± 0.18	785
	Collagen	27.1 ± 0.40	39.2 ± 0.06	49.7 ± 0.11	63.7 ± 0.23	85.7 ± 0.06	43.8 ± 0.35	20.5 ± 0.35	10.01
ZE-4c	AA	7.9 ± 0.15	15.4 ± 0.20	29 ± 0.21	43 ± 0.18	59 ± 0.03	75 ± 0.10	86.4 ± 0.44	55.3
	ADP	0.1 ± 0.03	2.7 ± 0.06	9.6 ± 0.15	22.5 ± 0.06	32 ± 0.12	39.7 ± 0.23	52.8 ± 0.12	850.4
	Collagen	33.5 ± 0.81	42.2 ± 0.24	50 ± 0.32	58.4 ± 0.32	68.4 ± 0.24	80.9 ± 0.26	85.9 ± 0.18	10
ZE-5a	AA	4.0 ± 0.12	7.9 ± 0.06	23.7 ± 0.15	39.5 ± 0.21	47.4 ± 0.12	68 ± 0.35	72.8 ± 0.59	121.6
	ADP	0.1 ± 0.09	1.8 ± 0.06	12.2 ± 0.12	24.3 ± 0.09	28.5 ± 0.12	36.3 ± 0.18	50.9 ± 0.17	956.8
	Collagen	23.3 ± 0.11	37.8 ± 0.49	43.3 ± 0.17	49.5 ± 0.23	67.6 ± 0.58	72.9 ± 0.46	81.4 ± 0.11	30.1
ZE-5b	AA	8.8 ± 0.09	11.4 ± 0.27	25 ± 0.21	30.7 ± 0.58	52.2 ± 0.40	68.4 ± 0.40	79 ± 0.60	99.9
	ADP	1 ± 0.03	3.6 ± 0.06	8.7 ± 0.17	22.5 ± 0.06	37.1 ± 0.14	44.9 ± 0.03	61.2 ± 0.17	519
	Collagen	21.6 ± 0.35	23.1 ± 0.41	43.8 ± 0.65	51.8 ± 0.43	67.8 ± 0.52	78.6 ± 0.31	91.1 ± 0.67	29.97
Aspirin	AA	27.2 ± 0.18	36 ± 0.09	50.1 ± 0.16	59.7 ± 0.09	100 ± 0	100 ± 0	100 ± 0	10.01
	ADP	3.6 ± 0.07	6.2 ± 0.09	19.1 ± 0.07	25 ± 0.06	32.8 ± 0.10	49.8 ± 0.12	56.9 ± 0.18	308.4
	Collagen	37.2 ± 0.14	48.7 ± 0.14	57.7 ± 0.20	68.6 ± 0.29	71 ± 0.23	78.6 ± 0.23	98.1 ± 0.11	3.2

Values are shown as mean of % platelet aggregation inhibition ± SEM, n = 3–4

#### Inhibitory effect on ADP-induced platelet aggregation

At 1, 3, 10, 30, 100, 300 and 1000 µM concentrations of the test compounds, ZE-4b inhibited platelet aggregation to 0.1 ± 0.03, 1.0 ± 0.03, 3.6 ± 0.03, 9.6 ± 0.06, 18.2 ± 0.12, 39.4 ± 0.17 and 54.7 ± 0.18% respectively with IC_50_ value of 785 µM. ZE-4c inhibited platelet aggregation to 0.1 ± 0.03, 2.7 ± 0.06, 9.6 ± 0.15, 22.5 ± 0.06, 32 ± 0.12, 39.7 ± 0.23 and 52.8 ± 0.12% respectively with IC_50_ value of 850.4 µM. The antiplatelet effect of ZE-5a was observed to be 0.1 ± 0.09, 1.8 ± 0.06, 12.2 ± 0.12, 24.3 ± 0.09, 28.5 ± 0.12, 36.3 ± 0.18 and 50.9 ± 0.17% respectively with IC_50_ value of 956.8 µM. ZE-5b inhibited platelet aggregation to 1 ± 0.03, 3.6 ± 0.06, 8.7 ± 0.17, 22.5 ± 0.06, 37.1 ± 0.14, 44.9 ± 0.03 and 61.2 ± 0.17% respectively with IC_50_ value of 519 µM. Aspirin exhibited inhibition of 3.6 ± 0.07, 6.2 ± 0.09, 19.1 ± 0.07, 25 ± 0.06, 32.8 ± 0.10, 49.8 ± 0.12 and 56.9 ± 0.18% respectively with IC_50_ value of 308.4 µM as presented in Table 1.

#### Inhibitory effect on collagen-induced platelet aggregation

The test compounds were evaluated for collagen-induced platelet aggregation inhibition at concentrations of 1, 3, 10, 30, 100, 300 and 1000 µM. ZE-4b showed inhibition of 27.1 ± 0.40, 39.2 ± 0.06, 49.7 ± 0.11, 63.7 ± 0.23, 85.7 ± 0.06, 43.8 ± 0.35 and 20.5 ± 0.35% respectively with IC_50_ value of 10.01 µM. ZE-4c inhibited platelet aggregation to 33.5 ± 0.81, 42.2 ± 0.24, 50 ± 0.32, 58.4 ± 0.32, 68.4 ± 0.24, 80.9 ± 0.26 and 85.9 ± 0.18% respectively with IC_50_ value of 10 µM. ZE-5a inhibited to 23.3 ± 0.11, 37.8 ± 0.49, 43.3 ± 0.17, 49.5 ± 0.23, 67.6 ± 0.58, 72.9 ± 0.46 and 81.4 ± 0.11% respectively with IC_50_ value of 30.1 µM. The inhibitory effect of ZE-5b was 21.6 ± 0.35, 23.1 ± 0.41, 43.8 ± 0.65, 51.8 ± 0.43, 67.8 ± 0.52, 78.6 ± 0.31 and 91.1 ± 0.67% respectively with the IC_50_ value of 29.97 µM. Aspirin inhibited platelet aggregation to 37.2 ± 0.14, 48.7 ± 0.14, 57.7 ± 0.20, 68.6 ± 0.29, 71 ± 0.23, 78.6 ± 0.23 and 98.1 ± 0.11% respectively with IC_50_ value of 3.2 µM as presented in Table 1.

### Anticoagulant assay

#### Effect on PRT

**Fig. 2:**
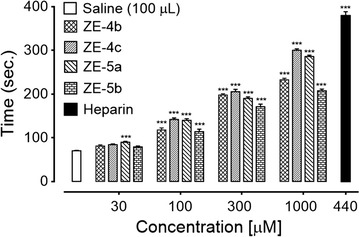
Bar chart showing increase in plasma recalcification time by different concentrations of *N*-[{(2-phenyl)methylidene]-2-(4-ethyl-5-(pyridine-2-yl)-4*H*-1,2,4-triazole-3-yl)sulfanyl}acetohydrazide (ZE-4b), *N*-[{(2-phenyl)methylidene]-2-(4-(fluorophenyl-5-(pyridine-2-yl)-4*H*-1,2,4-triazole-3yl)sulfanyl}acetohydrazide (ZE-4c), *N*-[{(4-methylphenyl)sulfonyl}]-2-(4-cyclohexyl-5-(pyridine-2-yl)-4*H*-1,2,4-triazole-3-yl)sulfanyl}acetohydrazide (ZE-5a), *N*-[{(4-methylphenyl)sulfonyl}-2-(4-ethyl-5-(pyridine-2-yl)-4*H*-1,2,4-triazole-3yl)sulfanyl} aceto-hydrazide (ZE-5b) and heparin. Data expressed as mean ± SEM, n = 5, ***P < 0.001 vs. saline group, one way ANOVA with post hoc Tukey’s test

The synthesized derivatives ZE-4(b–c) and ZE-5(a–b) were tested for their anticoagulant effect at different concentrations of 30, 100, 300 and 1000 µM. ZE-4b increased coagulation time to 81.40 ± 2.58, 118.2 ± 4.53, 197.8 ± 3.17 and 232.8 ± 3.41 s (P < 0.001 vs. saline group) respectively. ZE-4c increased coagulation time to 84.2 ± 1.88, 142 ± 3.51, 205.6 ± 5.37 and 300.2 ± 3.48 s (P < 0.001 vs. saline group) respectively. In case of ZE-5a coagulation time increased to 89.8 ± 2.35, 139.8 ± 3.93, 190.2 ± 3.65 and 286 ± 2.98 s (P < 0.001 vs. saline group) respectively. Similarly ZE-5b also increased the coagulation time to 79.2 ± 2.27, 114.2 ± 5.39, 171.4 ± 5.93, 207.6 ± 3.92 s (P < 0.001 vs. saline group) respectively. Heparin, at 440 µM concentration, increased coagulation time to 379.4 ± 9.18 s (Fig. 2).

#### Effect on BT

**Fig. 3:**
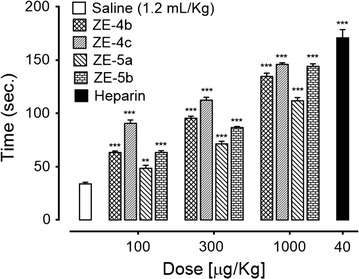
Bar chart showing increase in tail bleeding time by different doses of *N*-[{(2-phenyl)methylidene]-2-(4-ethyl-5-(pyridine-2-yl)-4*H*-1,2,4-triazole-3-yl)sulfanyl}acetohydrazide (ZE-4b), *N*-[{(2-phenyl)methylidene]-2-(4-(fluorophenyl-5-(pyridine-2-yl)-4*H*-1,2,4-triazole-3yl)sulfanyl}acetohydrazide (ZE-4c), *N*-[{(4-methylphenyl)sulfonyl}]-2-(4-cyclohexyl-5-(pyridine-2-yl)-4*H*-1,2,4-triazole-3-yl)sulfanyl}acetohydrazide (ZE-5a), *N*-[{(4-methylphenyl)sulfonyl}-2-(4-ethyl-5-(pyridine-2-yl)-4*H*-1,2,4-triazole-3yl)sulfanyl}acetohydrazide (ZE-5b) and heparin in mice. Data expressed as mean ± SEM, n = 4, **P < 0.01, ***P < 0.001 vs. saline group, one way ANOVA with post hoc Tukey’s test

The effect of test compounds ZE-4(b–c) and ZE-5(a–b) on bleeding time (BT) was studied at dose levels of 100, 300 and 1000 µM. ZE-4b increased BT to 63.25 ± 1.31, 95.25 ± 2.01 and 134.5 ± 3.122 s (P < 0.001 vs. saline group) respectively. ZE-4c increased BT to 90.5 ± 3.12, 112.25 ± 2.66 and 145.75 ± 1.60 s (P < 0.001 vs. saline group) respectively. In case of ZE-5a bleeding time increased to 48.25 ± 2.92, 71.25 ± 2.56 and 111.75 ± 3.04 s (P < 0.001 vs. saline group) respectively. ZE-5b increased BT to 63.25 ± 1.65, 86.5 ± 1.04 and 144 ± 2.38 s (P < 0.001 vs. saline group) respectively. Heparin, at 30 µM dose, increased BT to 170.75 ± 7.75 s (Fig. 3).

### Docking evaluation

**Fig. 4:**
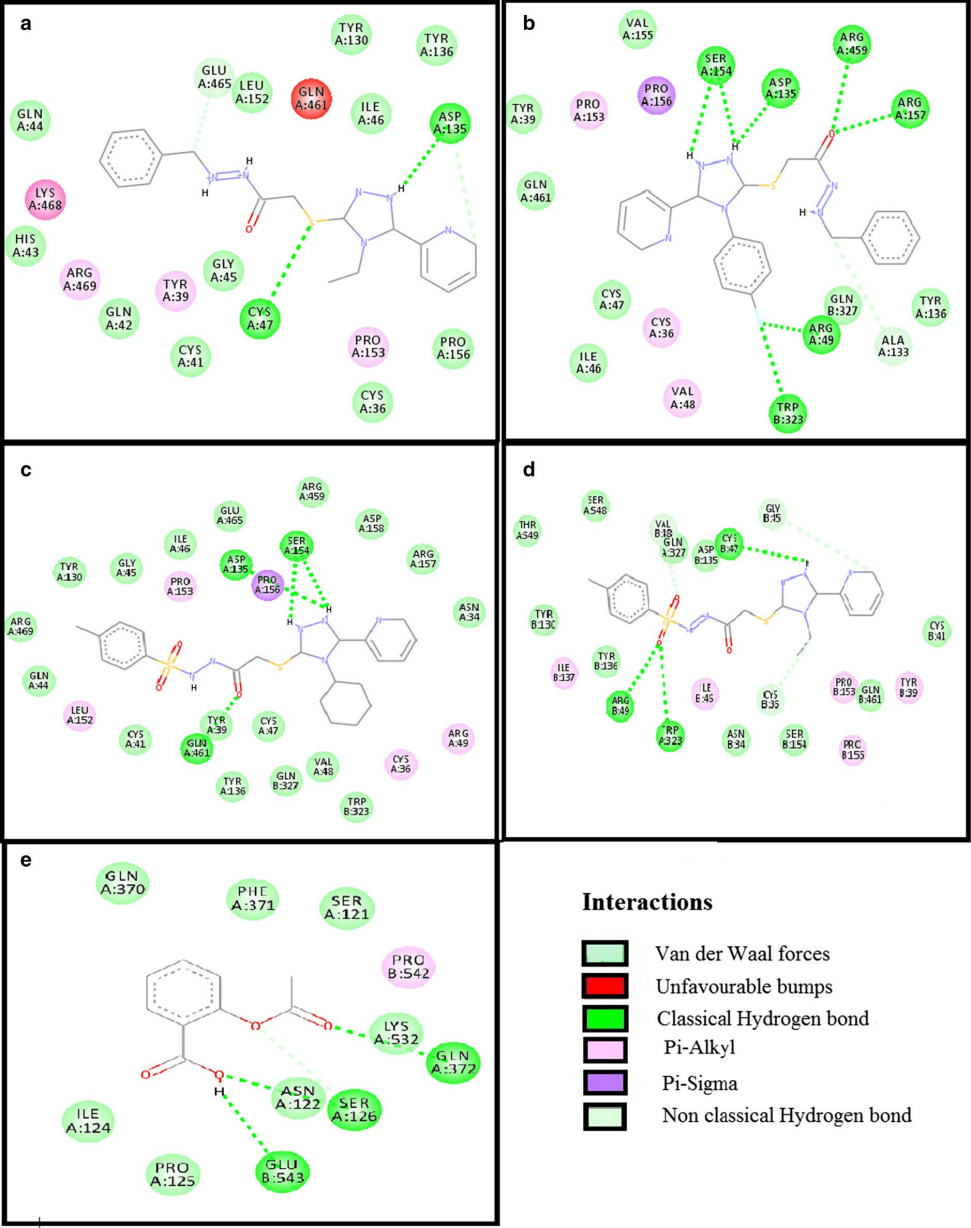
**a**–**e** Represent interactions of ligands: *N*-[{(2-phenyl)methylidene]-2-(4-ethyl-5-(pyridine-2-yl)-4*H*-1,2,4-triazole-3-yl)sulfanyl}acetohydrazide (ZE-4b), *N*-[{(2-phenyl)methylidene]-2-(4-(fluorophenyl-5-(pyridine-2-yl)-4*H*-1,2,4-triazole-3yl)sulfanyl}acetohydrazide (ZE-4c), *N*-[{(4-methylphenyl)sulfonyl}]-2-(4-cyclohexyl-5-(pyridine-2-yl)-4*H*-1,2,4-triazole-3-yl)sulfanyl}acetohydrazide (ZE-5a), *N*-[{(4-methylphenyl)sulfonyl}-2-(4-ethyl-5-(pyridine-2-yl)-4*H*-1,2,4-triazole-3yl)sulfanyl}acetohydrazide (ZE-5b) and aspirin respectively with target cyclooxygenase-1 (COX-1), drawn through Discovery Studio Visualizer client 2016

**Fig. 5:**
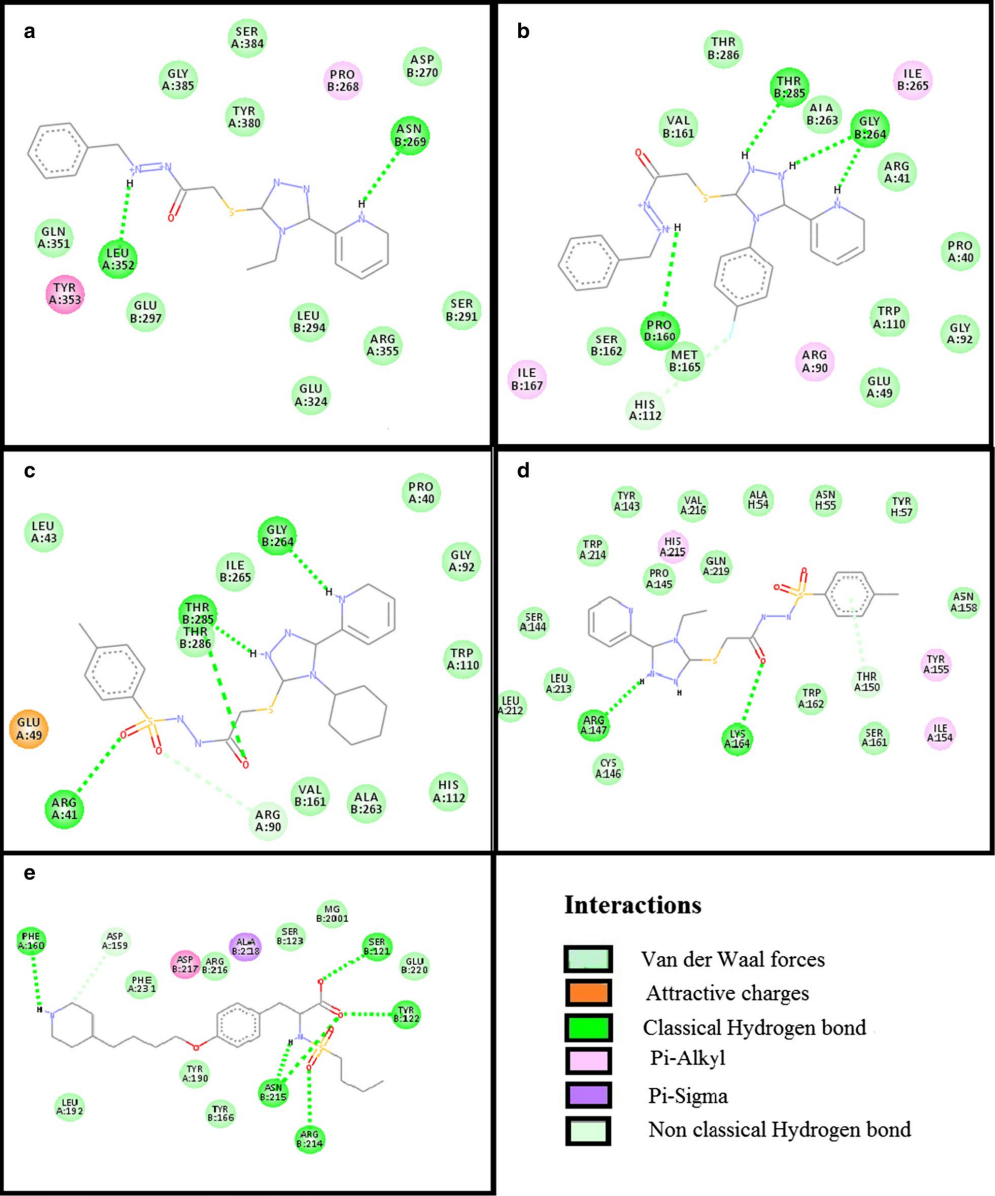
**a**–**e** Represent interactions of ligands: *N*-[{(2-phenyl)methylidene]-2-(4-ethyl-5-(pyridine-2-yl)-4*H*-1,2,4-triazole-3-yl)sulfanyl}acetohydrazide (ZE-4b), *N*-[{(2-phenyl) methylidene]-2-(4-(fluorophenyl-5-(pyridine-2-yl)-4*H*-1,2,4-triazole-3yl)sulfanyl}acetohydrazide (ZE-4c), *N*-[{(4-methylphenyl)sulfonyl}]-2-(4-cyclohexyl-5-(pyridine-2-yl)-4*H*-1,2,4-triazole-3-yl)sulfanyl}acetohydrazide (ZE-5a), *N*-[{(4-methylphenyl)sulfonyl}-2-(4-ethyl-5-(pyridine-2-yl)-4*H*-1,2,4-triazole-3yl)sulfanyl}acetohydrazide (ZE-5b) and tirofiban respectively with target glycoprotein IIb/IIIa (GP-IIb/IIIa), drawn through Discovery Studio Visualizer client 2016

**Fig. 6:**
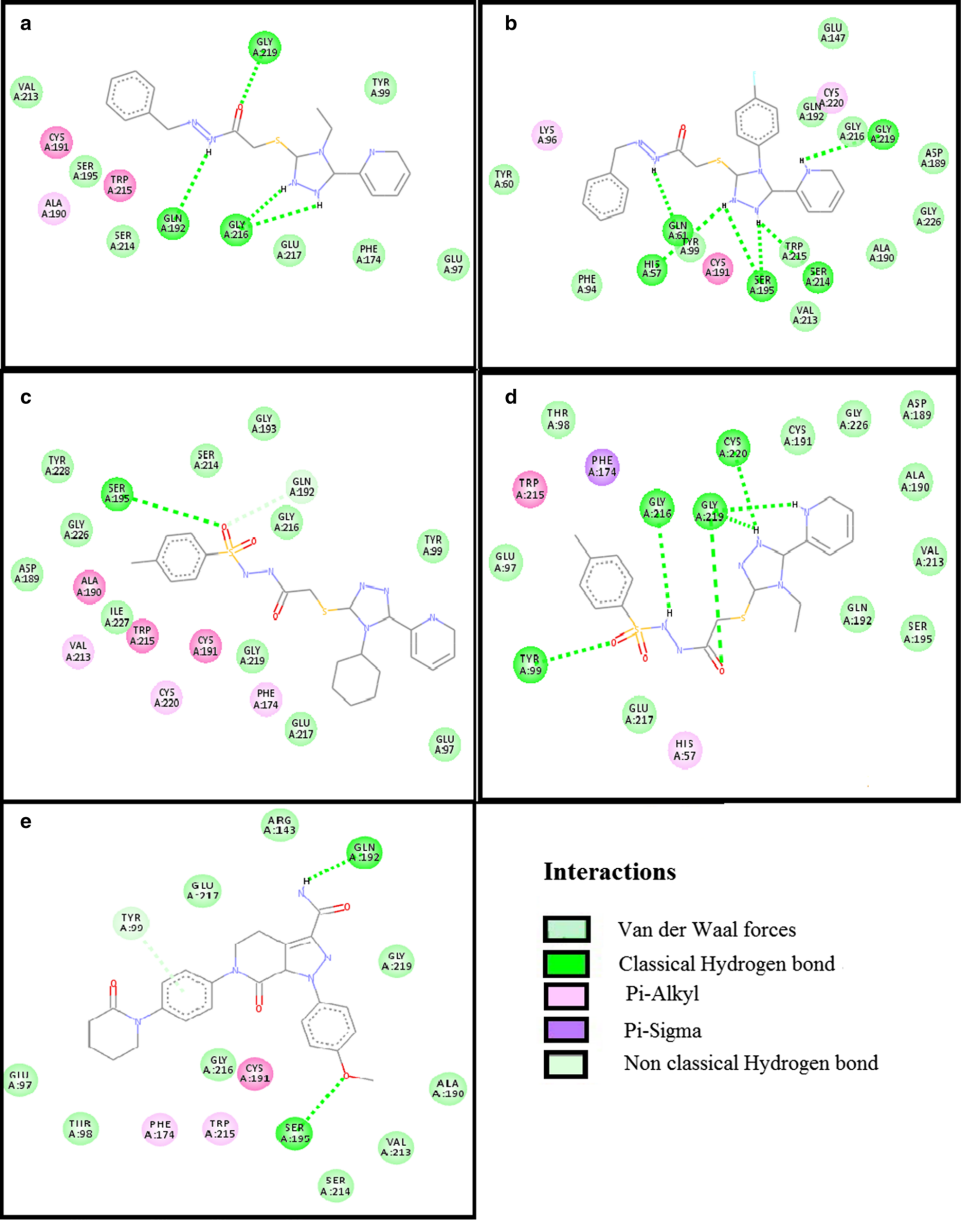
**a**–**e** Represent interactions of ligands: *N*-[{(2-phenyl)methylidene]-2-(4-ethyl-5-(pyridine-2-yl)-4*H*-1,2,4-triazole-3-yl)sulfanyl}acetohydrazide (ZE-4b), *N*-[{(2-phenyl)methylidene]-2-(4-(fluorophenyl-5-(pyridine-2-yl)-4*H*-1,2,4-triazole-3yl)sulfanyl}aceto-hydrazide (ZE-4c), *N*-[{(4-methylphenyl)sulfonyl}]-2-(4-cyclohexyl-5-(pyridine-2-yl)-4*H*-1,2,4-triazole-3-yl)sulfanyl}acetohydrazide (ZE-5a), *N*-[{(4-methylphenyl)sulfonyl}-2-(4-ethyl-5-(pyridine-2-yl)-4*H*-1,2,4-triazole-3yl)sulfanyl}acetohydrazide (ZE-5b) and apixaban respectively with target factor-X (F-X), drawn through Discovery Studio Visualizer client 2016

Test compounds showed variable affinities for different platelet and coagulant targets. Against COX-1, ZE-4b, ZE-4c, ZE-5a, ZE-5b and aspirin showed E-value of − 10.4, − 10.6, − 10.1, − 9.3 and − 6.1 kcal/mol respectively. 2D-interaction diagrams showing hydrogen bonds of ZE-4b, ZE-4c, ZE-5a, ZE-5b and aspirin with COX-1 are presented in Fig. 4. ZE-4b, ZE-4c, ZE-5a, ZE-5b and tirofiban against GP-IIb/IIIa showed E-value of − 8.6, − 9.9, − 9.9, − 8.7 and − 7.9 kcal/mol respectively. 2D-interaction showing hydrogen bonds of ZE-4b, ZE-4c, ZE-5a, ZE-5b and tirofiban with GP-IIb/IIIa receptor are shown in Fig. 5. Against GP-VI, ZE-4b, ZE-4c, ZE-5a, ZE-5b and hinokitiol showed E-value of − 6.4, − 7.3, − 7.2, − 6.9 and − 5.8 kcal/mol respectively. Against P_2_Y_12_ receptor, ZE-4b, ZE-4c, ZE-5a, ZE-5b and clopidogrel (active metabolite) showed E-value of − 6.8, − 6.9, − 5.8, − 7.4 and − 8.0 kcal/mol respectively. Against PG-I_2_ receptor, ZE-4b, ZE-4c, ZE-5a, ZE-5b and beraprost showed E-value of − 6.8, − 7.5, − 8.1, − 8.5 and − 8.3 kcal/mol respectively. Against PAR-1 receptor, ZE-4b, ZE-4c, ZE-5a, ZE-5b and vorapaxar showed E-value of − 6.5, − 7.9, − 8.5, − 7.7 and − 12.4 kcal/mol respectively. Against AT-III receptor, ZE-4b, ZE-4c, ZE-5a, ZE-5b and heparin sulfate showed E-value of − 6.6, − 8.1, − 8.4, − 8.3 and − 4.1 kcal/mol respectively. Against F-X, ZE-4b, ZE-4c, ZE-5a, ZE-5b and apixaban showed E-value of − 8.4, − 10.1, − 8.2, − 8.3 and − 9.2 kcal/mol respectively. 2D interaction, showing hydrogen bonds of ZE-4b, ZE-4c, ZE-5a, ZE-5b and apixaban with F-X are shown in Fig. 6. Against F-II, ZE-4b, ZE-4c, ZE-5a, ZE-5b and argatroban showed E-value of − 7.1, − 8.0, − 7.4, − 7.9 and − 8.0 kcal/mol respectively. Against F-IX, ZE-4b, ZE-4c, ZE-5a, ZE-5b and pegnivacogin showed E-value of − 8.4, − 8.1, − 7.2, − 7.8 and − 9.6 kcal/mol respectively. Against VKOR, ZE-4b, ZE-4c, ZE-5a, ZE-5b and warfarin showed E-value of − 7.8, − 8.3, − 8.3, − 7.2 and − 12.4 kcal/mol respectively. The best-docked poses of ligand–protein complex, having maximum binding energy values, no of hydrogen bonds (classical and non-classical) and residues involved in hydrogen bonding are summarized in Tables 2 and 3.

**Table 2:** E-value (kcal/mol) and post-docking analysis of best pose of *N*-[{(2-phenyl)methylidene]-2-(4-ethyl-5-(pyridine-2-yl)-4*H*-1,2,4-triazole-3-yl)sulfanyl}acetohydrazide (ZE-4b), *N*-[{(2-phenyl) methylidene]-2-(4-(fluorophenyl-5-(pyridine-2-yl)-4*H*-1,2,4-triazole-3yl)sulfanyl}acetohydrazide (ZE-4c), *N*-[{(4-methylphenyl)sulfonyl}]-2-(4-cyclohexyl-5-(pyridine-2-yl)-4*H*-1,2,4-triazole-3-yl)sulfanyl}acetohydrazide (ZE-5a) and *N*-[{(4-methylphenyl)sulfonyl}-2-(4-ethyl-5-(pyridine-2-yl)-4*H*-1,2,4-triazole-3yl)sulfanyl}acetohydrazide (ZE-5b) with cyclooxygenase-1 (COX-1), glycoprotein-IIb/IIIa (GP-IIb/IIIa), glycoprotein-VI (GP-VI), purino receptor P_2_Y_12_, prostacyclin receptor (PG-I_2_) and protein activated receptor-1 (PAR-1)

Targets	ZE-4b			ZE-4c			ZE-5a			ZE-5b			Standard drugs			
	E-value	H-bonds	Bonding residues	E-value	H-bonds	Bonding residues	E-value	H-bonds	Bonding Residues	E-value	H-bonds	Bonding residues	Standard	E-value	H-bonds	Bonding residues
COX-1	− 10.4	4	CYS 47 ASP 135(2) GLU 465	− 10.6	8	SER 154(2) ASP 135 ARG 459 ARG 157 ALA 133 ARG 49 TRP 323	− 10.1	4	SER 154(2) ASP 135 GLN 461	− 9.3	5	GLY 45 CYS 47 VAL 48 ARG 49 TRP 323	Aspirin	− 6.1	4	ASN 122 SER 126 LYS 532 GLU 543
GP-IIb/IIIa	− 8.6	2	ASN 269 LEU 352	− 9.9	5	HIS 112 PRO 160 GLY 264(2) THR 285	− 9.9	5	ARG 41 ARG 90 THR 285(2) GLY 264	− 8.7	3	ARG 147 THR 150 LYS 164	Tirofiban	− 7.9	7	SER 121 TYR 122 ASP 159 PHE 160 ARG 214 ASN215(2)
GP-VI	− 6.4	7	GLY 101 PRO102(2) ALA 103 VAL104(2) ASP 109	− 7.3	3	THR 157 THR 157 GLU 179	− 7.2	7	GLY 101(2) PRO 102(2) VAL 104(2) GLY 108	− 6.9	9	ARG 38 ARG 67 SER 69(4) TRP 76 SER77(2)	Hinokitiol	− 5.8	1	SER16
P_2_Y_12_	− 6.8	4	ASN 58 ASP121(2) GLN 124	− 6.9	2	ASN 65 VAL 146	− 5.8	1	ASN 65	− 7.4	3	ASN 65 VAL 146(2)	Clopidogrel (A.Metab)	− 8.0	4	SER 113(2) ASN201(2)
PG-I_2_	− 6.8	5	GLY 32 HIS 33 ASP 64 GLU 66 LYS 65	− 7.5	3	SER 10 GLY 32 GLU 66	− 8.1	4	HIS 33 HIS 68 SER 111(2)	− 8.5	5	HIS 33(2) LEU 34 HIS 68(2)	Beraprost	− 8.3	2	ARG 36 HIS 74
PAR-1	− 6.5	3	GLY1030 ASP 1070 GLN 1105.	− 7.9	2	ASN 1020 GLU 1022	− 8.5	5	LEU 258 GLU 260 HIS 336 SER 344(2)	− 7.7	3	ASP 256 LEU 258 SER 344	Vorapaxar	− 12.4	6	ASP 256 VAL 257 LEU 258 TYR 337 ALA349(2)

(2), 2 hydrogen bonds with the same residue; GLN, glutamine; CYS, cysteine; ARG, arginine; TYR, tyrosine; SER, serine; GLU, glutamic acid; TRP, tryptophan; ALA, alanine; THR, threonine; HIS, histidine; ASN, asparagine; VAL, valine; LYS, lysine; GLY, glycine; PHE, phenylalanine; ASP, aspartic acid

**Table 3:** E-value (kcal/mol) and post-docking analysis of best pose of *N*-[{(2-phenyl)methylidene]-2-(4-ethyl-5-(pyridine-2-yl)-4*H*-1,2,4-triazole-3-yl)sulfanyl}acetohydrazide (ZE-4b), *N*-[{(2-phenyl) methylidene]-2-(4-(fluorophenyl-5-(pyridine-2-yl)-4*H*-1,2,4-triazole-3yl)sulfanyl}acetohydrazide (ZE-4c), *N*-[{(4-methylphenyl)sulfonyl}]-2-(4-cyclohexyl-5-(pyridine-2-yl)-4*H*-1,2,4-triazole-3-yl)sulfanyl}acetohydrazide (ZE-5a) and *N*-[{(4-methylphenyl)sulfonyl}-2-(4-ethyl-5-(pyridine-2-yl)-4*H*-1,2,4-triazole-3yl)sulfanyl}acetohydrazide (ZE-5b) with antithrombin-III (AT-III), factor-X (F-X), factor-II (F-II), factor-IX (F-IX) and vitamin-K epoxide reductase (VKOR)

Targets	ZE-4b			ZE-4c			ZE-5a			ZE-5b			Standard drugs			
	E-value	H-bonds	Bonding residues	E-value	H-bonds	Bonding residues	E-value	H-bonds	Bonding residues	E-value	H-bonds	Bonding residues	Standard	E-value	H-bonds	Bonding residues
AT-III	− 6.6	4	LYS 241(2) GLY 244 PRO 288	− 8.1	4	ALA 143, ASN 144(2) G LU 163	− 8.4	5	SER 291(2) ASP 172(2) GLY 244	− 8.3	4	ASP 149 ASP 360 ASP 361(2)	Heparin SO_4_	− 4.1	6	ASN 233 GLN268(2) VAL 388 ARG393(2)
F-X	− 8.4	4	GLN 192 GLY 21(2) GLY 219	− 10.1	6	HIS 57 GLN 61 SER 195(2) SER 214 GLY 219	− 8.2	2	GLN 19 SER 195	− 8.3	6	TYR 99 GLY 216 GLY219(3) CYS 220	Apixaban	− 9.2	3	TYR 99 GLN 192 SER 195
F-II	− 7.1	3	GLU 14C SER 203 ASN 205	− 8.0	2	ARG 126 LYS 236	− 7.4	6	TRP 60D TRP 96(2) ARG 97 TYR 60A GLU 97A	− 7.9	6	THR128(2) SER203 ASP125(2) TYR 208	Argatroban	− 8.0	7	GLU 39 LEU 40 LEU 41, ASN 143 GLU 192 THR 147B ALA 147C
F-IX	− 8.4	5	ALA 56(2) HIS 57 THR 601 TYR 94	− 8.1	3	HIS 57 TYR 99 SER 214	− 7.2	2	SER 15 SER 214	− 7.8	5	CYS 58 TYR 99(2) SER 195 SER 214	Pegnivacogin	− 9.6		NA
VKOR	− 7.8	5	THR 34(2) LEU 60 MET 111 CYS 133	− 8.3	2	SER 61 ASP 214	− 8.3	2	GLY 76 LEU 107	− 7.2	4	LYS 41 GLU 44 SER 61(2)	Warfarin	− 12.4	2	THR 34 LYS 41

NA, not available; (2), 2 hydrogen bonds with the same amino acid residue; GLN, Glutamine; CYS, cysteine; ARG, arginine; TYR, tyrosine; SER, serine; GLU, glutamic acid; TRP, tryptophan; ALA, alanine; THR, threonine; HIS, histidine; ASN, asparagine; VAL, valine; LYS, lysine; GLY, glycine; PHE, phenylalanine; ASP, aspartic acid

## Discussion

A series of six new 1,2,4-triazole derivatives were synthesized by following Scheme 1. Among these were three hydrazone ZE-4(a–c) and three sulphonamide derivatives ZE-5(a–c). All these were characterized by spectroscopic techniques including FTIR, ^1^HNMR, ^13^CNMR and elemental analysis data. All the synthesized derivatives were obtained in good yields except ZE-4a and ZE-5c. The compounds obtained in good yields were evaluated for their antiplatelet and anticoagulant potential using different in silico, in vitro and in vivo assays. To assess the antiplatelet potential, three different agonists were used. In AA induced platelet aggregation, test derivatives showed concentration dependent inhibition. The order of test compounds for platelet aggregation inhibition was as ZE-4b > ZE-4c > ZE-5b > ZE-5a. It is also observed that 1,2,4-triazole hydrazone derivatives i.e. ZE-4b and ZE-4c showed better activity than 1,2,4-triazole sulphonamide derivatives. The possible reason could be the presence of *N*-acyl hydrazone (NAH) moiety. NAH subunit can increase the antiplatelet potential of compounds because of its high affinity and inhibitory activity for COX-1 resulting in greater inhibition of TXA_2_ formation [21]. It can also decrease the concentration of intracellular calcium by acting as a calcium chelator and thus can interfere with platelet activation and aggregation [22]. We can infer that ZE-4b and ZE-4c may have inhibited the COX-1 receptor like aspirin, resulting in decreased production of TXA2 and thus inhibition of platelet aggregation [23]. This is also supported by high affinity of test compounds for COX-1. In ADP-induced platelet aggregation, test compounds did not show any significant inhibition, even at a higher dose of 1000 µM, showing that these derivatives did not interfere significantly with ADP receptors like P_2_Y_12_. In collagen-induced platelet aggregation assay, test compounds exhibited significant inhibition with order of inhibition as ZE-4c > ZE-4b > ZE-5b > ZE-5a. This inhibitory effect clearly indicated the effect of test compounds on collagen receptors i.e. GP-IIb/IIIa or VI [24]. Test compounds have also shown high affinity for GP-IIb/IIIa in docking study, so it is possible that these derivatives interfere the binding of fibrinogen to GP-IIb/IIIa receptor and consequently aggregation of platelets [25]. The synthesized compounds ZE-4(**b**–**c**) and ZE-5(a–b) were further investigated for their anticoagulant action via two different models. The test compounds increased PRT and BT with ZE-4c being most effective, which could be attributed to the presence of NAH subunit as it depletes the intracellular calcium by acting as calcium chelator and thus inhibiting the coagulation process [26]. The presence of aromatic *p*-fluorophenyl substitution at N-4 of triazole ring enhanced the anticoagulant effect of ZE-4c [27]. In molecular docking study, ZE-4c have shown high binding energy for F-X.

## Conclusions

In the present study, six new 1,2,4-triazole derivatives ZE-4(a–c) and ZE-5(a–c) were synthesized. ZE-4b, ZE-4c, ZE-5a and ZE-5b were obtained in good yield and further evaluated for their antiplatelet and anticoagulant potential. The test compounds showed antiplatelet activity less than the standard drug, however, hydrazone derivatives ZE-4b and ZE-4c were found to be more potent as compared to sulphonamide derivatives. ZE-4c also exhibited potent anticoagulant activity by increasing PRT and BT time. Further, the molecular interactions of test compounds were investigated by molecular docking studies against selected targets of blood aggregation and coagulation pathways. Test compounds possessed high affinity for COX-1, GP-IIb/IIIa and F-X receptors. The in vitro and in vivo studies also confirmed antiplatelet and anticoagulant potential of test compounds.

## Abbreviations

ADP: adenosine diphosphate
AA: arachidonic acid
COX-1: cyclooxygenase-1
GP-IIb/IIIa: glycoprotein-IIb/IIIa
GP-VI: glycoprotein-VI
PAR-1: protein activated receptor-1
AT-III: antithrombin-III
PRT: plasma recalcification time
BT: bleeding time
PDB: protein data bank
TXA2: thromboxane-A2
NAH: *N*-acyl hydrazone
